# P-1281. A Novel Substance Use Disorder-Focused Infectious Diseases Consultation Service: Our Experience with Xylazine-Related Ulcers

**DOI:** 10.1093/ofid/ofae631.1462

**Published:** 2025-01-29

**Authors:** Elizabeth Novick, Carolyn Kramer, John Zurlo, Dagan Coppock, Nikhil Seval, Mollie Tucker

**Affiliations:** Thomas Jefferson University Hospital, Philadelphia, Pennsylvania; Thomas Jefferson University Hospital, Philadelphia, Pennsylvania; Jefferson University, Hershey, PA; Thomas Jefferson University Hospital, Philadelphia, Pennsylvania; Drexel University College of Medicine, Philadelphia, Pennsylvania; Thomas Jefferson University, Philadelphia, Pennsylvania

## Abstract

**Background:**

Xylazine is a non-opioid analgesic that has become a prevalent additive in the illicit drug supply due to its low cost and synergistic effect with opioids. It is associated with soft tissue ulcers that become necrotic and can lead to infection, amputation, and death. Our hospital created a novel Infectious Diseases (ID) consultation service to help manage xylazine-related ulcers and other infectious sequelae of substance use disorder (SUD). Herein we describe characteristics of patients with SUD who presented to our hospital with xylazine-related soft tissue ulcers.

**Figure:**
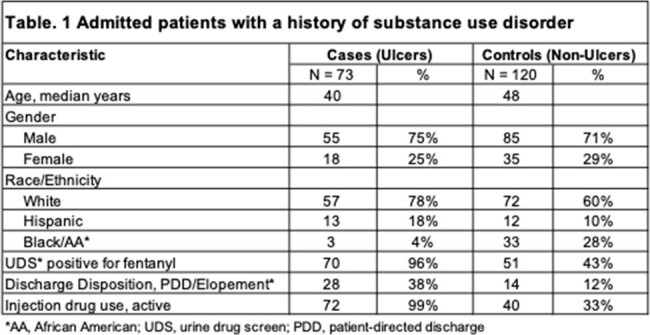

**Methods:**

Using data extracted from our electronic health record, we generated a database of patients seen on our ID-SUD service between October 10, 2023 and January 1, 2024. We identified patients ≥ 18 years with documented SUD and conducted a retrospective chart review to identify patients with ulcers. Ulcers were defined based on documentation within the physical examination of a provider’s note or under the photographic media section. Patient demographics and clinical characteristics were collected and described using simple counts and frequencies.

**Figure:**
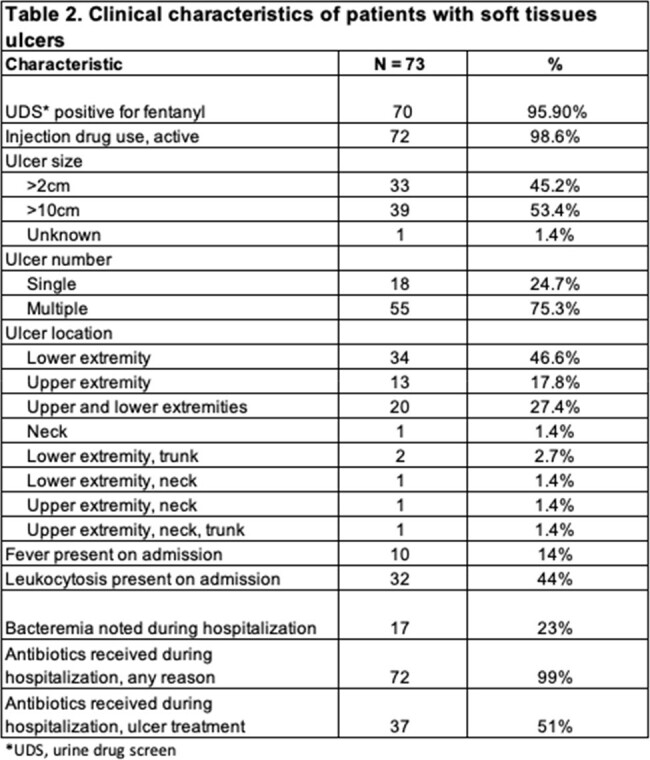

**Results:**

We identified 193 patients with SUD seen by the ID consult team during the study period. 73 patients (38%) had skin ulcers. The ulcer group was younger with a median age of 40, more likely to be white (78%) or Hispanic (18%), more likely to have tested positive for fentanyl (96%), more likely to inject (99%), and more likely to leave the hospital against medical advice (38%) compared to the non-ulcer patients, as shown in Table 1. Of patients with ulcers, 39 (53%) had ulcers >10cm in size, 55 (75%) had multiple ulcers, 10 (14%) had fever on admission, 17 (23%) developed bacteremia during hospitalization, and 72 (99%) received antibiotics during hospitalization, as shown in Table 2.

**Conclusion:**

Xylazine-related skin ulcers have become common in patients with SUD, in particular intravenous opioid use. In our adult population in a large city with high rates of SUD, we demonstrated that many patients develop multiple large ulcers. Additionally, many patients with ulcers had evidence of systemic infection with fever, leukocytosis, and/or bacteremia. We anticipate an improvement in clinical outcomes as more is learned about pathophysiology and treatment of xylazine-related ulcers.

**Disclosures:**

**All Authors**: No reported disclosures

